# Characterising pharmacist interventions from person-centred medicines reviews in general practice settings

**DOI:** 10.1016/j.rcsop.2026.100833

**Published:** 2026-08-18

**Authors:** Ciara Kirke, Kevin Murphy, Laura J. Sahm, Frank Moriarty, Clare Kinahan, Leon O'Hagan, Emma J. Coyle, Stephen Byrne, Kieran Dalton

**Affiliations:** a Health Service Executive, Ireland; b National Medication Safety Programme, National Quality and Patient Safety Directorate, Health Service Executive, Ireland; c Pharmaceutical Care Research Group, School of Pharmacy, University College Cork, Cork, Ireland; d School of Pharmacy and Biomolecular Sciences, RCSI University of Medicine and Health Sciences, Dublin, Ireland; e Rotunda Hospital, Parnell Square, Dublin, Ireland

**Keywords:** General practice, Medication review, Patient participation, Pharmacist, Polypharmacy, Prescribing, Primary care

## Abstract

**Background:**

With pharmacist roles in general practices not widely implemented, it is not clear what interventions are undertaken as part of patient-centred medicines reviews in that setting.

**Objectives:**

To describe the types of general practice pharmacist interventions from person-centred medicines reviews with patients with hyperpolypharmacy (on ≥10 regular medicines) and/or at high risk of medicines-related harm, and evaluate their significance, rates of acceptance and implementation, and resulting changes in medicines numbers.

**Methods:**

Four pharmacists were integrated into ten general practice settings, who conducted medicines reviews following the ‘*7 Steps to Appropriate Polypharmacy*’ approach, and liaised with general practitioners, other healthcare professionals, and patients to make review-related interventions and follow up. Intervention significance was classified using Eadon grading. General practitioner acceptance and implementation of recommendations to alter prescriptions was recorded. Descriptive and inferential statistics were performed on summary data.

**Results:**

Between January 2021 and December 2022, 1471 patients consented to data collection (mean age: 76 years, 88.3% with hyperpolypharmacy). A mean of twelve interventions/patient was made. For patients with complete intervention data (*n* = 1181), 38.7% of 14,592 interventions were recommendations to alter prescriptions; of these with known outcomes (*n* = 5537), 95% were accepted and 82.7% implemented. Prescription alterations comprised stops (55.9%), decreased doses (19.0%), starts (18.9%), and increased doses (6.2%). Nearly all interventions were deemed ‘significant’ (98.4%); 29% of patients had ≥1 ‘very significant’ intervention, of which 75% related to stopping a medicine. Patients had a mean of 13.8 pre-review medicines and 12.3 post-review medicines (mean difference − 1.5 [SD 2.0], *p* < 0.0001), with an 11.2% reduction in medicines and 11.6% fewer patients with hyperpolypharmacy post review.

**Conclusion:**

Pharmacist interventions from person-centred medicines reviews in general practice were highly accepted, generating substantial medicines reductions and improved care for patients at high risk of medicines-related harm. Future research should explore the longer-term sustainment of these reviews in more diverse healthcare contexts.

## Highlights

• In-depth account of general practice pharmacists' person-centred medicines reviews.
• Large study (*n* = 1471) including patients at higher risk of medicines-related harm.
• Nearly all of pharmacists' prescription recommendations were accepted.
• A wider variety of pharmacist interventions compared to most medicines reviews.
• An average of 12 pharmacist interventions and 1.5 fewer medicines per patient.

## 1 Introduction

As the prevalence of multimorbidity rises with age, multiple medicines are often prescribed to manage each of the co-morbid conditions. This means that polypharmacy and hyperpolypharmacy (typically defined where patients are on ≥5 and ≥ 10 regular medicines respectively) are highly prevalent, especially in older adults.^1^, ^2^ A systematic review and meta-analysis by Wang et al. found that polypharmacy and hyperpolypharmacy were present in 39.1% and 13.3% of older adults worldwide.^3^ Polypharmacy is often required to help manage multimorbidity, and can be appropriate where there is a favourable risk-benefit balance in line with patient preferences.^4^ However, polypharmacy is sometimes considered a ‘necessary evil’,^5^ as it is associated with potentially inappropriate prescribing, which can increase the risk of adverse events (e.g., falls), functional decline, and healthcare utilization.^6^, ^7^ Polypharmacy is associated with significantly higher healthcare expenditure which is not just related to increased medicines alone, as it also augments healthcare practitioner visits and inpatient hospital stays.^8^, ^9^ Polypharmacy can also be a burden which negatively affects patients' quality of life, as well as resulting in poor medicines adherence,^10^, ^11^, ^12^ and it is also associated with a higher risk for mortality.^13^ It is thus no surprise that the World Health Organization (WHO), as part of their third Global Patient Safety Challenge ‘*Medication Without Harm*’, highlighted polypharmacy as a priority area for action to reduce medication-related harm.^14^

To address this, medicines reviews by pharmacists with patients with polypharmacy have been shown to be beneficial.^15^ It can be more challenging for community pharmacists to perform such reviews on a large scale, due to factors such as healthcare records access, time constraints, and lack of remuneration.^16^ There has been a rise in the number of jurisdictions where pharmacists work in general practice settings, with a wide range of activities that include, but are not limited to, medicines reconciliation, medicines review, patient education, addressing queries from fellow healthcare professionals, and prescribing (both initiating and repeating).^17^ Evidence indicates that general practice pharmacists can decrease both the number of medicines (by approximately 1 medicine per patient) and potentially inappropriate prescribing, as well as reducing general practitioner (GP) workload and emergency department hospitalisations by up to 47%.^18^, ^19^

Despite the reported benefits, having pharmacists in general practice or similar primary care settings is not commonplace in many jurisdictions, including Ireland, often due to regulatory barriers, financial constraints, and infrastructure limitations.^20^ However, an opportunity arose to integrate pharmacists into Irish general practices as part of routine care delivery through the European Union-funded “implementing Stimulating Innovation in the Management of Polypharmacy and Adherence Through the Years” (iSIMPATHY) project.^21^ The most recent Irish data showed that hyperpolypharmacy prevalence was 21.9% in people aged ≥65 years qualifying for public health cover in 2012, which increased from 1.5% in 1997.^22^ This was higher than the 13.3% reported by Wang et al..^3^ The iSIMPATHY initiative aimed to improve the health and well-being of people with hyperpolypharmacy and those at higher risk of medicines-related harm by having pharmacists lead comprehensive person-centred medicines reviews. In Ireland, these iSIMPATHY medicines reviews were delivered in general practices, and it has already been shown that they were both cost-effective and clinically effective (i.e., improved medicines appropriateness and patient-reported outcomes, which included adherence and quality of life).^23^, ^24^ Given that the global evidence for pharmacist interventions in general practices is still at a relatively early stage, it is important to characterise the interventions that pharmacists have performed in successful initiatives like iSIMPATHY. This will not only help with evidence-based refinement of current practices in jurisdictions with general practice pharmacists, it will also highlight the net benefit in jurisdictions without it and potentially aid integrating pharmacist models internationally. It will also provide a greater understanding of the medicines-related issues that are prevalent in these patient groups. Therefore, this study's objectives were to:

(i) quantify and characterise the types and clinical significance of pharmacist interventions,
(ii) describe the medicines classes involved in these interventions,
(iii) evaluate the acceptance and implementation rates of interventions involving recommendations to change prescriptions, and
(iv) evaluate the number of medicines changes (overall and per patient).

## 2 Methods

### 2.1 Ethics approval and reporting

Advice was obtained from the Health Service Executive Dublin North East Research Ethics Committee. Under national guidance, the work met service evaluation criteria,^25^ so ethics committee approval was not required. The project complied fully with data protection requirements and information governance, including patient consent for data collection, processing, and analysis. The tool to enhance pharmacist patient care intervention reporting (PaCIR) and the Template for Intervention Description and Replication (TIDieR) checklist guided study reporting.^26^, ^27^

### 2.2 Study context, design, and participants

The iSIMPATHY project involved conducting medicines reviews with people at higher risk of medicines-related harm in Scotland, Northern Ireland, and Ireland. For this, reviews were performed in general practice and hospital settings (inpatient and outpatient), the findings of which have been previously reported.^21^ As reviews were not performed in hospital settings in Ireland, this was a single arm pre-post study comprising a secondary analysis to characterise the interventions undertaken by pharmacists in iSIMPATHY general practices in Ireland from January 2021 to December 2022 where patients consented to data processing and met ≥1 of the following inclusion criteria:

1. Prescribed ≥10 regular medicines.
2. At greater risk of medicines-related harm, as defined by the presence of ≥1 polypharmacy indicator.^28^
3. Adults of any age, approaching end-of-life due to any cause.
4. Aged ≥50 years and living in a residential care setting.

General practices in Ireland (near the Northern Ireland border) were invited to express interest in participating in the iSIMPATHY project. Ten general practices were selected so that each pharmacist (*n* = 4) was linked with approximately 20,000 patients, ensuring sufficient complex patients likely to avail of medicines reviews in the timeline. Three pharmacists were employed for 37 h/week, working in 1, 2 and 3 GP practices respectively, and one in two GP practices for 20 h/week. When the study started, pharmacists had 7, 13, 15, and 20 years' post-registration experience in community pharmacy (*n* = 2), community pharmacy and primary care (*n* = 1), and community and hospital pharmacy (*n* = 1), respectively.

In line with the funding body's requirements, all review-related activity for each eligible patient was considered one review (i.e., data from one review per patient was recorded).

### 2.3 Training and quality assurance

A comprehensive 28-h training programme enabled project pharmacists to deliver medicines reviews, apply Scottish Polypharmacy Guidance,^29^ and complete data collection and project evaluation tools, with a quality assurance process to aid consistent categorisation (described elsewhere).^21^ Throughout the project, education, training, support, and peer-to-peer learning (including case discussions and peer mentoring) was provided.

### 2.4 Medicines review delivery

Pharmacists identified eligible patients using the practice software or through referrals from GPs or other healthcare professionals. The pharmacist telephoned the patient to explain the service and ask if they wished to be reviewed. Information about data collection and processing was provided and consent sought verbally (due to COVID-19 restrictions). Patients who did not consent were still eligible for a medicines review, with only their unique project identifier and gender recorded.

Pharmacists prepared for reviews by reviewing the patient's medical history, medicines history, and laboratory results as relevant. Reviews were initially conducted over the telephone, due to COVID-19 restrictions. Patients could choose a face-to-face appointment or telephone review once restrictions were eased. The ‘*7 Steps to appropriate polypharmacy*’ approach was used as a guide to structure the review process with patients (and their nominated carer where applicable).^29^ The pharmacist asked “*What matters?*” to the patient initially. The pharmacist and patient then consider whether each medication is, or has the potential to be, appropriate, necessary, effective, harmful, and sustainable/cost-effective. Thereafter, the review concludes with an agreed and shared medication plan. The person-centred nature of the review allowed tailoring of interventions to individual needs.

Pharmacists implemented interventions where possible (e.g., medicines reconciliation, education provision, and referral for monitoring) and, where appropriate, liaised with other healthcare professionals involved in patients' care (e.g., in community pharmacies and specialist clinics). When a review was complete, the pharmacist provided a written report of recommendations for the patient's GP to assess and action. Protected time was booked for GPs to review reports, where possible. A fee was paid to GPs for each review.

Follow-up usually took place 2–6 weeks post review (up to 12 weeks), depending on the intervention type or complexity and the time needed to assess and resolve. This involved the pharmacist assessing outcomes of interventions from reviews, monitoring, and a discussion by telephone with the patient (or their nominated carer), where possible and appropriate.

### 2.5 Data collection and analysis

Summary data were recorded on an electronic spreadsheet for all patients consenting to data collection. This consisted of age, gender, number of comorbidities, number of review interventions, number of medicines pre and post review, and time spent on review activities (including follow-up).

Data on patients' medicines, comorbidities, and result of interventions had to be recorded for at least 50% of each pharmacist's reviews; data could be recorded for more patients if time permitted. Medicines were recorded and classified according to the WHO Anatomical Therapeutic Chemical (ATC) Classification at the ATC 3rd level.^30^ All comorbidities included in a list of 40 common comorbidities (provided by iSIMPATHY project organisers) were recorded (Appendix 1). Any interventions recorded had their clinical significance graded by the pharmacist making the intervention, using grading described by Eadon^31^ and the result of the intervention was recorded at follow-up. Two intervention types had to be recorded for all consenting patients:

- Eadon grade 5 (i.e., ‘very significant’) or above interventions.^31^
- interventions involving polypharmacy indicators; information on the polypharmacy indicators present in this patient group is reported elsewhere.^24^

Within-class drug switches were recorded as drug stops and starts; a sub-analysis was performed to identify these from the data.

Descriptive statistics were generated for the result categories of interventions and the medicine involved and the number and percentage of interventions graded in each Eadon category reported. The percentage acceptance and implementation rates for interventions were calculated based on known outcomes at the time of project follow-up. A specific sub-analysis of the ‘very significant’ interventions characterised the intervention result and medicines involved. A two-tailed student's *t*-test was performed to check for differences in the number of medicines pre and post review.

## 3 Results

### 3.1 Patient population

Between January 2021 and December 2022, 2217 patients received iSIMPATHY reviews in Ireland. Of the 1906 (86%) patients who consented to data collection, 1471 met ≥1 of the inclusion criteria:

- Prescribed ≥10 regular medicines: *n* = 1299 (88.3%)
- Had ≥1 polypharmacy indicator: *n* = 693 (47.1%)
- Approaching end-of life: *n* = 125 (8.5%)
- Care home resident aged >50 years: *n* *=* 39 (2.7%)

Participants had a mean age of 76.0 years (standard deviation [SD] 9.5; range 35–101), with 90.1% aged ≥65 years, whilst 55.1% were female and 44.9% male.

A mean of 6.2 comorbidities (SD 2.3) was recorded. The frequency of specific comorbidities recorded for 1049 patients (71.3%) is included in Appendix 1. Cardiovascular comorbidities were the most common, with hypertension (62.6%), coronary heart disease (48.2%), atrial fibrillation (28.3%), and heart failure (16.7%) all featuring in the top 10.

### 3.2 Number of medicines and hyperpolypharmacy prevalence

**Fig. 1:**
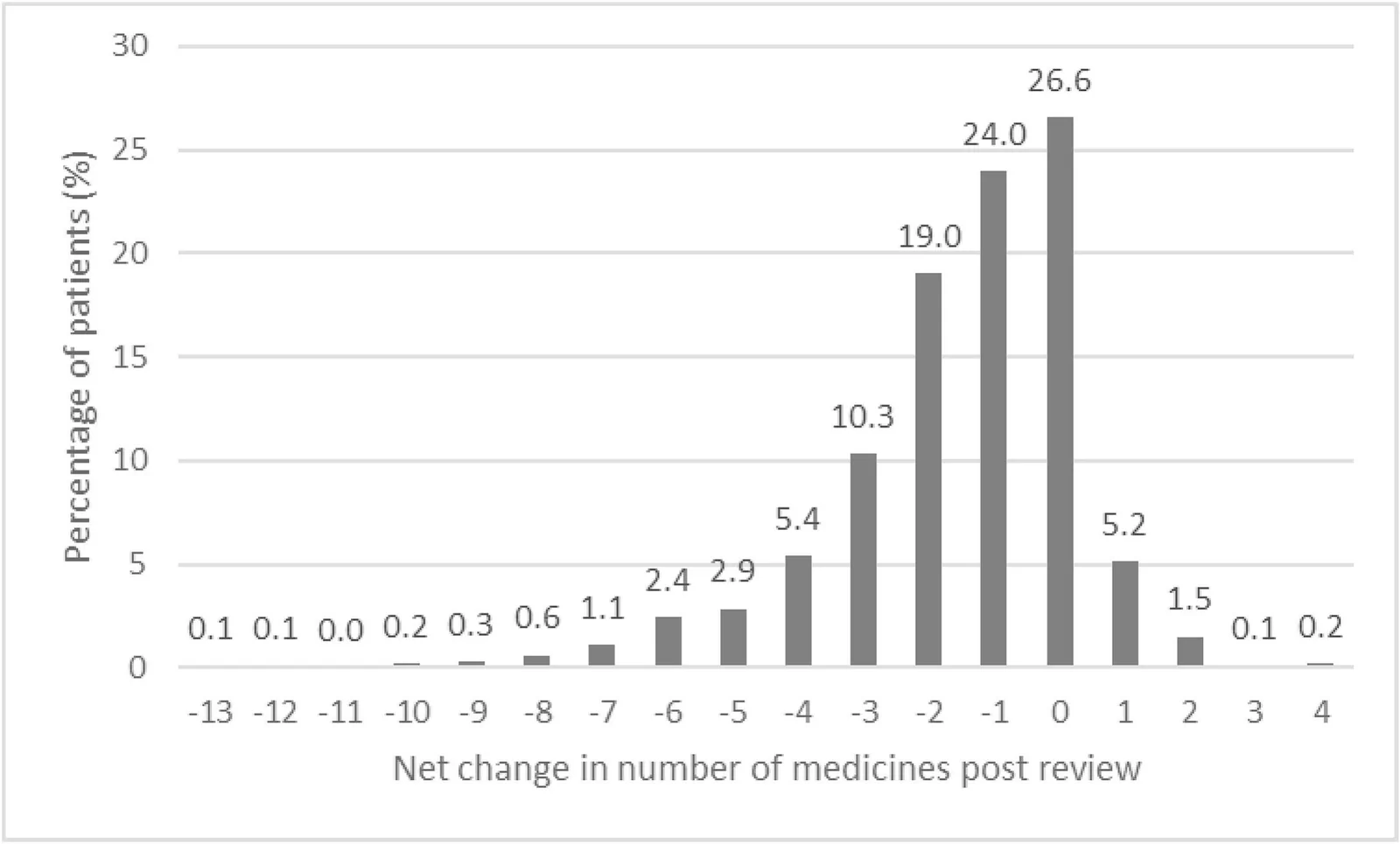
Net change in number of medicines post review (*n* = 1471).

Patients were prescribed a mean of 13.8 medicines pre review (SD 4.7) and 12.3 medicines (SD 4.3) post review. The mean difference post versus pre review was −1.5 (SD 2.0, 95% confidence interval − 1.2 to −1.9, *p* < 0.0001), with a range of four additional medicines to 13 fewer medicines post review. The net reduction was 2276 medicines, i.e., a net decrease of 11.2% of the total medicines prescribed at baseline. A net reduction in the number of medicines was achieved in 66.5% of patients (*n* = 978), whilst 7.0% (*n* = 102) had a net increase (Fig. 1).

Hyperpolypharmacy (≥10 medicines) was present in 1299 patients (88.3%) pre review and in 1129 (76.8%) post review, i.e., resulting in 170 fewer people with hyperpolypharmacy post review (11.6% of patients reviewed).

### 3.3 Time per review

Excluding data collection, which took a mean of 13.5 min (SD 8.8), total pharmacist time spent on reviews was a mean of 157.2 min (SD 45.6), which comprised a mean of:

- 61.6 min (SD 16.2) on pre-review preparation,
- 36.5 min (SD 11.0) undertaking the review with the patient (and nominated carer, where applicable), and
- 59.1 min (SD 29.7) post review on actioning recommendations, communicating with the GP and other healthcare professionals, and patient follow-up.

GPs spent a mean of 16.6 min (SD 7.4) on review-related activities, primarily reviewing and actioning recommendations post review.

### 3.4 Pharmacist interventions

A total of 17,600 interventions were made, a mean of 12.0 per patient (SD 4.2; range 3–29). Full intervention result categories were recorded for 14,592 interventions (82.9%), which are presented in Table 1 and relate to 1181 patients (80.3% of patients, mean 12.4/patient).

**Table 1:** Pharmacist intervention result categories for patients with full intervention data (*n* = 1181).

Intervention result categories	Number	% of interventions	Mean per patient
**Recommendation to alter prescription**	**5652**	**38.7%**	**4.8**

Prescription altered	4578	31.4%	3.9
- Drug stopped	2560	17.5%	2.2
- Dose decreased	870	6.0%	0.7
- Drug started	866	5.9%	0.7
- Dose increased	282	1.9%	0.2
Prescription unchanged at time of data collection	1074	7.4%	0.9
- Recommendation accepted, but not implemented	684	4.7%	0.6
- Recommendation not accepted and not implemented	275	1.9%	0.2
- Not implemented, acceptance status unknown	115	0.8%	0.1

**Information or education provided**	**4121**	**28.2%**	**3.5**

Patient/carer education	2093	14.3%	1.8
Information shared with another healthcare professional	1255	8.6%	1.1
Information shared with patient	773	5.3%	0.7

**Monitoring or referral**	**2595**	**17.8%**	**2.0**

Requested/reviewed test/investigation			
- Implemented	1692	11.6%	1.4
- Not implemented	211	1.4%	0.2
Referral made			
- Implemented	644	4.4%	0.5
- Not implemented	48	0.3%	0.04

**Medicines reconciliation-related**	**1489**	**10.2%**	**1.3**

Medicines reconciliation	1181	8.1%	1
Prescription endorsed	220	1.5%	0.2
Patient's own drugs reviewed			
- Implemented	81	0.6%	0.1
- Not implemented	7	0.05%	0.006

**Other**	**735**	**5%**	**0.6**

- Implemented	728	5%	0.6
- Not implemented	7	0.05%	0.006

**Total**	**14,592**	**100%**	**12.4**

Table 1 shows that a mean of 4.8 recommendations to alter prescriptions were made per patient, with a mean of 3.9 changes made per patient. There were 5537 recommendations to alter prescriptions with known outcomes (i.e., excluding recommendations with unknown outcomes or not implemented by the time of data collection); of these, 95.0% were accepted (*n* = 5262), 82.7% accepted and implemented (*n* = 4578), whilst 12.4% were accepted but not implemented (*n* = 684).

### 3.5 Significance of pharmacist interventions

Table 2 shows the intervention significance grading in the 1181 patients with full intervention data – corresponding to 82.7% of interventions (*n* = 14,552), nearly all of which were graded as being positively clinically significant (i.e., grades ≥3; 98.4%), including 3.7% (*n* = 536) graded as ‘very significant’, having prevented a major organ failure or adverse reaction of similar importance (Eadon grade 5).

**Table 2:** Eadon grading of intervention significance in patients with full intervention data (*n* = 1181).

Eadon grade^31^	Number	% of graded Interventions
1. Detrimental to patient	0	–
2. Of no significance to patient	230	1.6%
3. Significant: does not improve patient care	2348	16.1%
4. Significant: improves patient care	11,438	78.6%
5. Very significant: prevents a major organ failure or adverse reaction of similar importance	536⁎	3.7%
6. Potentially lifesaving	0	–

**Total**	**14,552**†	**100%**

⁎ This excludes the additional 45 ‘very significant’ interventions recorded for patients without full intervention data.

† A small portion of interventions did not have their significance recorded unintentionally.

An additional 45 ‘very significant’ interventions were recorded for patients without full recording of intervention data, corresponding to 581 interventions in total relating to 427 patients (i.e., 29% of included patients had ≥1 ‘very significant’ intervention).

### 3.6 Drug classes

Of the 5537 recommendations to alter prescriptions with known outcomes, details pertaining to the drug class (as per WHO ATC classification) are provided for 4866 of these (87.9%) in Appendix 2; this is because some recommendations were not specific to one drug class. From these recommendations, the twenty most common drug classes addressed in this study are presented in Table 3 (as per WHO ATC classification), while Appendix 3 includes recommendations for 350 within-class switches, where one drug was stopped and another drug started within the same medicines class (defined based on the WHO ATC 3rd level code). Drugs most commonly switched, which accounted for over half of all switches, were:

- those for peptic ulcer and gastro-oesophageal reflux disease (20.0%, *n* = 70),
- blood glucose lowering drugs excluding insulins (13.7%; *n* = 48),
- lipid modifying agents (12.2%; *n* = 43), and
- antithrombotics (11.4%; *n* = 40).

**Table 3:** Top 20 medicines classes where a prescription alteration recommendation was made (*n* = 1181).

ATC 3rd level Classification		Prescription altered (*n*)				Prescription not altered (*n*)				
Code	Code descriptor	Drug stop	Dose decrease	Drug start	Dose increase	Recommendation accepted	Recommendation not accepted	Total	% Accepted	% Altered
A02B	Drugs for peptic ulcer and gastro-oesophageal reflux disease	121	202	120	2	34	14	493	97.2	90.3
C10A	Lipid modifying agents, plain	114	38	97	38	35	13	335	96.1	85.7
A10B	Blood glucose lowering drugs, excluding insulins	152	41	74	33	24	10	334	97.0	89.8
B01A	Antithrombotic agents	190	20	51	8	23	10	302	96.7	89.1
N06A	Antidepressants	76	40	30	13	25	8	192	95.8	82.8
M01A	Antiinflammatory and antirheumatic products, non-steroids	170	7	5	1	4	3	190	98.4	96.3
B03B	Vitamin B12 and folic acid	123	1	20	2	4	3	153	98.0	95.4
N02B	Other analgesics and antipyretics	34	33	34	34	14	1	150	99.3	90.0
R03A	Adrenergics, inhalants	64	8	56	1	3	10	142	93.0	90.8
N02A	Opioids	75	24	15	3	13	2	132	98.5	88.6
C07A	Beta blocking agents	51	45	11	7	8	3	125	97.6	91.2
A11C	Vitamin A and D, including combinations of the two	48	11	57	4	2	1	123	99.2	97.6
C08C	Selective calcium channel blockers with mainly vascular effects	58	16	10	8	15	3	110	97.3	83.6
G04B	Urologicals	72	15	10	3	7	2	109	98.2	91.7
A06A	Drugs for constipation	66	15	13	8	4	2	108	98.1	94.4
C09A	ACE inhibitors, plain	42	17	18	11	5	2	95	97.9	92.6
B03A	Iron preparations	65	3	13	5	5	2	93	97.8	92.5
C03C	High-ceiling diuretics	49	21	10	3	8	1	92	98.9	90.2
A12A	Calcium	46	6	26	3	4	1	86	98.8	94.2
N05C	Hypnotics and sedatives	19	34	3	1	17	10	84	88.1	67.9

### 3.7 ‘Very significant’ interventions

Details regarding the intervention category for all ‘very significant’ (i.e., Eadon grade 5) interventions are displayed in Table 4. Most ‘very significant’ interventions pertained to prescription changes (94.7%), and the majority related to stopping a drug (75.6% of total). Table 4 also shows the five most common drug classes involved in stops, starts, and dose decreases for these interventions. The drug classes involved across all ‘very significant’ interventions are shown in Appendix 4.

**Table 4:** ‘Very significant’ interventions, including the 5 most common drug classes for some categories (*n* = 581, involving 427 patients).

Intervention category	Number	%
Drug stopped	439	75.6
- Anti-inflammatory and antirheumatic products, non-steroids	116	
- Antithrombotic agents	113	
- Blood glucose lowering drugs, excluding insulins	21	
- Antidepressants	16	
- Beta blocking agents	15	

Drug started	54	9.3
- Antithrombotic	17	
- Drugs for peptic ulcer and gastro-oesophageal reflux diseas	16	
- Lipid modifying agents, plai	5	
- Calcium	3	
- Blood glucose lowering drugs, excluding insulins	2	

Dose decreased	52	9.0
- Beta blocking agent	10	
- Antithrombotic agent	8	
- Antidepressant	4	
- Blood glucose lowering drugs, excluding insulins	3	
- Angiotensin-converting enzyme inhibitors, plain	3	

Referral made	9	1.5

Information shared with another healthcare professional	7	1.2

Other (e.g., removing items from medicines list, prescription endorsed)	7	1.2

Dose increased	5	0.9

Requested/reviewed test/investigation/measurement	4	0.7

Patient/carer education or information	4	0.7

## 4 Discussion

This study demonstrates the variety, scale, and significance of interventions that general practice pharmacists can make when conducting comprehensive person-centred medicines reviews. There were 12 pharmacist interventions made per patient on average, including drug change recommendations, education, monitoring, and referrals. A mean of 4.8 recommendations to alter prescriptions were made per patient, most of which were accepted (95%), whilst 82.7% were implemented in the project timeline. This resulted in nearly four prescription changes and 1.5 fewer medicines per patient on average, even accounting for medicines started. This represented an 11.2% reduction from medicines prescribed at baseline, alongside 11.6% fewer patients with hyperpolypharmacy post review. This is important because the number of medicines prescribed is strongly associated with inappropriate prescribing and medicines-related harm (including adverse drug reactions, reduced quality of life, and mortality).^32^, ^33^, ^34^, ^35^ The medicines reduction demonstrated in this study may be a key reason for the decrease in patient-reported side effects and impact of medicines on daily activities that were shown in a previous iSIMPATHY publication.^24^ These benefits, coupled with the other evidence from iSIMPATHY medicines reviews of enhanced medicines appropriateness, cost-effectiveness, and other improved patient-reported outcomes,^23^, ^24^ should be used as evidence to support the implementation of person-centred medicines reviews by primary care-based pharmacists in jurisdictions where they are not yet commonplace.^36^

Recommendations to alter prescriptions accounted for over one third of this study's recorded interventions (38.7%), with on average 4.8 per patient and 3.9 changes made per patient. This is higher than most studies report, with a mean of 3.6 drug-related problems reported per clinical medication review in Australian community settings.^37^ It is also higher than all but one study from a systematic review of pharmacist-led home medication reviews^38^; this exception found a mean of 7.4 treatment-related problems.^39^ However, the present study had a higher average of 12 interventions per patient from medicines reviews, and there may be several reasons for this. Comprehensive person-centred medicines reviews were not funded in the general practices prior to this study, possibly contributing to more intervention opportunities compared to practices where regular comprehensive reviews are resourced. Additionally, pharmacists in this study were funded to focus solely on conducting reviews as part of the iSIMPATHY project, so had sufficient time to conduct each review comprehensively, possibly allowing for more interventions to be made. Furthermore, many medicines review studies focus their interventions only on medicines changes, and do not detail additional interventions such as patient education and monitoring suggestions. Therefore, this study may better reflect the work that pharmacists undertake whilst comprehensively reviewing patients' medicines and thus should be considered when recording interventions in future research.

Beyond the multi-faceted intervention types, having patient involvement also enables more issues to be identified,^40^ as well as facilitating information sharing. In a previous general practice-based study in Ireland by Cardwell et al., pharmacists identified a mean of 1.93 prescribing issues per patient, which is substantially lower than this study. Among the study differences, 95.1% of the medicines reviews were conducted using patients' records only due to difficulty in recruiting patients for in-person reviews,^41^ rather than with patients in all reviews in the current study (which included reviews in person and via telephone). Anecdotally, patients in this study reported satisfaction with reviews over the phone due to not having to attend an appointment in person. Previous research has emphasised that patients' perspectives and preferences should be considered when dealing with polypharmacy.^42^, ^43^, ^44^ Some medicines optimisation opportunities can only be identified through such engagement,^45^ for example the patient's experience of undocumented adverse effects. The ‘*7 steps to appropriate polypharmacy*’ approach used in the iSIMPATHY reviews allowed pharmacists to establish what matters to the person, educate patients, engage in shared decision-making, and agreeing on a plan between the empowered patient and pharmacist; the steps and key principles from this review process have remained the same in the most recent Scottish Polypharmacy Guidance.^46^ Therefore, future reviews should aim to have such patient involvement – either in person or remotely (e.g., via telephone) – to help identify more medicines-related issues.

This study's volume of interventions was also influenced by its population; targeting patients with hyperpolypharmacy and at high risk of medicines-related harm increased the likelihood of having more medicines-related problems.^43^, ^47^, ^48^ While this targeting may affect the generalisibility of some findings, it is important to acknowledge that a realist review has identified that targeting high-risk patients is a key mechanism to enhance implementation of multidisciplinary medicines review and deprescribing in primary care.^43^ This and several other mechanisms cited were included in the present study, such as applying medicines review tools (e.g., explicit criteria such as the polypharmacy indicators), involving patients and carers in the process, and tailored follow-up plans.^43^ While these mechanisms may be more resource-intensive, the greater benefits (including substantial cost savings) from these comprehensive reviews mean that such mechanisms should be considered when designing similar future interventions. Resource-limited settings though may need to consider other approaches, such as narrower prioritisation of the highest-risk patients and sharing pharmacists across practices.

In this study, the GP acceptance rate of pharmacist recommendations for prescription changes was 95%. This compares well to acceptance rates of medicines optimisation interventions made by general practice pharmacists in previous studies of 88%,^49^ 86%,^50^ 75%.^51^ However, one in eight prescription change recommendations in this study (12.4%) were accepted but not implemented. Across the whole multi-jurisdictional iSIMPATHY project, 97% of non-implemented recommendations came from Ireland.^21^ Most iSIMPATHY pharmacists in Scotland and Northern Ireland were independent prescribers, whereas pharmacists in Ireland were dependent on GPs to make medicines changes. Reasons for non-implementation may have included not being able to implement the changes within the project timeline, GPs' time constraints,^52^ or forgetting to take action. Pharmacist prescriber status may increase the proportion of interventions implemented, and this is now in the process of being introduced in Ireland.^53^ Therefore, future interventions should consider prescribing capabilities where possible.

A systematic review of pharmacist-led primary care interventions to promote medicines optimisation in the United Kingdom found that recommendation implementation was strongly influenced by pharmacists' relationships with other health and care professionals, especially GPs.^54^ Pharmacists anecdotally perceived that this study's extended integration facilitated greater pharmacist-GP collaboration and the development of trust and co-operative working relationships. Personal acquaintance and developing interprofessional trust and rapport greatly facilitate physician implementation of pharmacist recommendations.^55^ A systematic review by Kwint et al. found a mean implementation rate of 50% for pharmacist recommendations by GPs; in examining the relationship between implementation rate and extent of pharmacist-GP collaboration, implementation rates of over 80% were seen in only 3/13 interventions, where six, seven, or eight ‘key elements’ were present.^42^ The present study had 82.7% of prescription change recommendations implemented and had six of these key elements described by Kwint et al.: (i) pharmacists with adequate clinical experience, (ii) a sharing of medical records, (iii) patient interview by pharmacist, (iv) invitation or referral for medicines review by the GP (practice), (v) an action plan, and (vi) follow-up. A further element, pharmacist-GP case conference, took place in some cases. The final element, own pharmacist involved, was not the case as the pharmacists were new to the practice and each patient only received one review and follow-up. Taking one element as an example: sharing of medical records allows the pharmacist to have similar patient information as the GP, aiding informed decision-making with recommendations, and making them more likely to be implemented. Given that studies with a high prescriber implementation rate of pharmacists' medication appropriateness recommendations are more likely to achieve significant improvements in patient outcomes,^56^, ^57^, ^58^, ^59^ this study's high implementation rate was likely a key factor in achieving substantial improvements in both medicines appropriateness and patient-reported outcomes.^24^ Therefore, the present study reinforces that the ‘key elements’ described should be considered when optimising interventions in future research and practice.

‘Very significant’ interventions judged to prevent a major organ failure or adverse reaction of similar importance (i.e., Eadon grade 5) were recorded in over one quarter of patients (*n* = 427; 29%), three quarters of which involved stopping medicines. Non-steroidal anti-inflammatory drugs (NSAIDs) and antithrombotics were not only the two most frequent drug classes recommended to be stopped, they also each represented over one quarter of these ‘very significant’ recommendations to stop medicines, corresponding to 26.4% and 25.7% respectively. Given that a systematic review found NSAIDs to be present in 6.2% of ADR-related hospital admissions^60^ and a recent Irish study found that antithrombotics were the drug class most frequently associated with ADR-related admissions,^61^ this supports the high significance rating and highlights drugs that should be prioritised for intervention in patients at high risk of medicines-related harm.

Substantial net cost savings were achieved by these reviews; this was shown in a previous publication under all modelling and sensitivity analyses performed – accounting for the cost of conducting the reviews relative to drug cost savings and avoiding both ADRs and hospital admissions.^23^ The intervention-based pharmacoeconomic model from that cost-benefit analysis calculated net cost savings of €651–€741 per review, with corresponding annual savings of €240,870–€457,197 per annum per pharmacist – even with the average review time of 157.2 min. Whilst the high number of interventions likely contributed to this review time, the pre-review preparation time was quite lengthy (mean: 61.6 min); pharmacists anecdotally reported that this was partly because medicines reconciliation was more complex and time-consuming than anticipated, as pharmacists adjusted the medicines-related details in practice records. Therefore, these review-related activities had other subsequent benefits not captured in the study evaluation; for example, these adjustments improved medicines information quality, which can aid medicines reconciliation and prescribing decisions, thus facilitating potential future impacts that include enhanced quality and efficiency of future reviews and prescribing practices. A recent Cochrane review concluded that medicines reviews combined with interventions including medicines reconciliation and patient education resulted in fewer hospital readmissions when compared to usual care, where standard medicines reviews did not.^62^ The iSIMPATHY project, however, did not evaluate these outcomes and this study's intervention outcomes were only recorded within 12 weeks post review. Whilst some recommendations were likely implemented after this time, some implemented recommendations may also have been reversed following data collection. However, a previous iSIMPATHY substudy found that 91% of dose decreases, 92% of stops, 95% of starts, and 100% of dose increases, made by pharmacist prescribers during iSIMPATHY reviews in Northern Ireland, were sustained to one year.^63^ Therefore, it is likely that a large proportion of this study's changes were also sustained longer term.

### 4.1 Strengths and limitations

This study's medicines reviews were delivered in a standardised way by four pharmacists across ten general practices. This study depicts a comprehensive overview of pharmacist interventions made across a two-year period, allowing time for pharmacist-GP relationship development and for the reviews to become part of routine care. Whilst data collection was completed up to 12 weeks after reviews, the lack of longer follow-up resulted in the outcome of some interventions, and long-term outcome of all interventions, not being captured.

While this study reports findings comparing post-review (post-intervention) data to pre-review (baseline) data, it is limited by its single-arm study design; future researchers should consider testing the review's effectiveness in a randomised controlled trial. In addition, there was no data collection nor analysis of those who did not consent; while this may have led to selection bias, it is estimated that any potential bias would be low given that almost 90% of those asked gave consent.

Within-drug class switches (*n* = 350) were recorded as 350 drug stops and 350 drug starts, in line with project data collection procedures. While this slightly increased the number of interventions recorded, these additional 350 interventions correspond to only 6.2% of prescription change recommendations and 2.4% of interventions overall.

Intervention recording and Eadon classification was completed for most patients, but not all, and classification and recording of this and all data was performed by the pharmacist conducting the review. Project pharmacists far exceeded the required minimum of 50% of their reviews in collecting this data, resulting in a large dataset. To further minimise the associated risk of bias in classifying interventions, standardised guidance, training, and quality assurance through peer review was provided.

## 5 Conclusion

This study has provided unique insights into the wide variety of interventions made as part of pharmacist-led person-centred medicines reviews in general practices. These reviews involved patients and were comprehensive in their approach, which resulted in a high number of pharmacist interventions per patient (*n* = 12) compared to previous studies. These iSIMPATHY medicines reviews comprised several key elements which are known to result in high acceptance and implementation rates of pharmacist interventions by GPs, which likely contributed to most interventions being accepted (95%) and implemented within the project timeline (82.7%). These key elements include patient interview by a clinically experienced pharmacist, shared medical records, and tailored follow-up plans. Most interventions were deemed to be significant in improving patient care, and these reviews generated a substantial net reduction in the number of medicines. Ultimately, the success of this initiative means that the approach used in these iSIMPATHY medicines reviews should strongly be considered for inclusion in the design and delivery of future medicines reviews services in primary care settings. It would be beneficial to explore how the intervention could be sustained in settings which have greater time constraints or are more resource-limited. Further examination of how to overcome potential barriers to implementation may help in translating these results widely to more diverse healthcare contexts.

## CRediT authorship contribution statement

**Ciara Kirke:** Writing – review & editing, Writing – original draft, Visualization, Project administration, Methodology, Investigation, Funding acquisition, Formal analysis, Data curation, Conceptualization. **Kevin Murphy:** Writing – review & editing, Visualization, Supervision, Methodology, Funding acquisition, Formal analysis, Data curation, Conceptualization. **Laura J. Sahm:** Writing – review & editing, Visualization, Methodology, Funding acquisition, Conceptualization. **Frank Moriarty:** Writing – review & editing, Visualization, Methodology, Funding acquisition, Formal analysis. **Clare Kinahan:** Writing – review & editing, Writing – original draft, Visualization, Methodology, Investigation, Formal analysis, Data curation, Conceptualization. **Leon O'Hagan:** Writing – review & editing, Writing – original draft, Methodology, Investigation, Formal analysis, Data curation, Conceptualization. **Emma J. Coyle:** Writing – review & editing, Writing – original draft, Methodology, Investigation, Data curation, Conceptualization. **Stephen Byrne:** Writing – review & editing, Methodology, Funding acquisition, Conceptualization. **Kieran Dalton:** Writing – review & editing, Writing – original draft, Visualization, Supervision, Project administration, Methodology, Funding acquisition, Formal analysis, Data curation, Conceptualization.

## Funding

The iSIMPATHY project was funded by the European Union INTERREG VA Programme (IVA 5081). The funders had no part in the study design, data collection, data analysis and interpretation, report writing, or the decision to submit the article for publication.

## Declaration of competing interest

The authors declare that they have no known competing financial interests or personal relationships that could have appeared to influence the work reported in this paper.

## Data Availability

The data associated with this study will not be made publicly available.
