# Supplementary material for: Characterising pharmacist interventions from person-centred medicines reviews in general practice settings
Source: Explor Res Clin Soc Pharm. 2026 Aug 18;24:100833. doi: 10.1016/j.rcsop.2026.100833 (PMC13602043; doi:10.1016/j.rcsop.2026.100833)
Supplement: Supplementary file 1 [file mmc1.docx]

# **Appendices**

# **Appendix 1: Specific comorbidities (*n*=1049 patients)**

| **Comorbidity** | **Number of patients** | **% of patients** |
| --- | --- | --- |
| Hypertension | 657 | 62.6% |
| Coronary heart disease | 506 | 48.2% |
| Painful condition | 418 | 39.8% |
| Diabetes | 377 | 35.9% |
| Atrial fibrillation | 297 | 28.3% |
| Treated dyspepsia | 213 | 20.3% |
| Chronic obstructive pulmonary disease | 199 | 19.0% |
| Heart failure | 175 | 16.7% |
| Chronic kidney disease | 155 | 14.8% |
| Rheumatoid arthritis, other inflammatory polyarthropathies and systemic connective tissue disorders | 139 | 13.3% |
| Stroke and/or transient ischaemic attack | 127 | 12.1% |
| Thyroid disorders | 124 | 11.8% |
| New diagnosis of cancer in last five years | 116 | 11.1% |
| Prostate disorders | 114 | 10.9% |
| Depression | 105 | 10.0% |
| Asthma (currently treated) | 97 | 9.2% |
| Diverticular disease of intestine | 97 | 9.2% |
| Anxiety and/or other neurotic, stress related or somatoform disorders | 84 | 8.0% |
| Blindness or low vision | 83 | 7.9% |
| Peripheral vascular disease | 76 | 7.2% |
| Glaucoma | 48 | 4.6% |
| Dementia | 45 | 4.3% |
| Psoriasis or eczema | 39 | 3.7% |
| Treated constipation | 35 | 3.3% |
| Inflammatory bowel disease | 32 | 3.1% |
| Irritable bowel syndrome | 24 | 2.3% |
| Parkinson's disease | 24 | 2.3% |
| Alcohol problems | 21 | 2.0% |
| Chronic liver disease | 19 | 1.8% |
| Hearing loss | 19 | 1.8% |
| Epilepsy (currently treated) | 17 | 1.6% |
| Schizophrenia (and related non-organic psychosis) or bipolar disorder | 16 | 1.5% |
| Chronic sinusitis | 14 | 1.3% |
| Bronchiectasis | 9 | 0.9% |
| Anorexia or bulimia | 6 | 0.6% |
| Learning disability | 5 | 0.5% |
| Migraine | 5 | 0.5% |
| Other psychoactive substance misuse | 2 | 0.2% |
| Multiple sclerosis | 1 | 0.1% |
| Viral hepatitis | 1 | 0.1% |

# **Appendix 2: Drug class associated with prescription change interventions for patients with full intervention data**

| **ATC 3^rd^ level Classification** | | **Prescription altered** | | | | **Prescription not altered** | |  |  |  |
| --- | --- | --- | --- | --- | --- | --- | --- | --- | --- | --- |
| **Code** | **Code descriptor** | **Drug stop** | **Drug start** | **Dose increase** | **Dose decrease** | **Advice accepted** | **Advice not accepted** | **Total** | **% Accepted** | **%**  **Altered** |
| A02B | Drugs for peptic ulcer and gastro-oesophageal reflux disease | 121 | 120 | 2 | 202 | 34 | 14 | 493 | 97.2 | 90.3 |
| C10A | Lipid modifying agents, plain | 114 | 97 | 38 | 38 | 35 | 13 | 335 | 96.1 | 85.7 |
| A10B | Blood glucose lowering drugs, excluding insulins | 152 | 74 | 33 | 41 | 24 | 10 | 334 | 97.0 | 89.8 |
| B01A | Antithrombotic agents | 190 | 51 | 8 | 20 | 23 | 10 | 302 | 96.7 | 89.1 |
| N06A | Antidepressants | 76 | 30 | 13 | 40 | 25 | 8 | 192 | 95.8 | 82.8 |
| M01A | Ant-inflammatory and anti-rheumatic products, non-steroids | 170 | 5 | 1 | 7 | 4 | 3 | 190 | 98.4 | 96.3 |
| B03B | Vitamin B12 and folic acid | 123 | 20 | 2 | 1 | 4 | 3 | 153 | 98.0 | 95.4 |
| N02B | Other analgesics and antipyretics | 34 | 34 | 34 | 33 | 14 | 1 | 150 | 99.3 | 90.0 |
| R03A | Adrenergics, inhalants | 64 | 56 | 1 | 8 | 3 | 10 | 142 | 93.0 | 90.8 |
| N02A | Opioids | 75 | 15 | 3 | 24 | 13 | 2 | 132 | 98.5 | 88.6 |
| C07A | Beta blocking agents | 51 | 11 | 7 | 45 | 8 | 3 | 125 | 97.6 | 91.2 |
| A11C | Vitamin A and D, including combinations of the two | 48 | 57 | 4 | 11 | 2 | 1 | 123 | 99.2 | 97.6 |
| C08C | Selective calcium channel blockers with mainly vascular effects | 58 | 10 | 8 | 16 | 15 | 3 | 110 | 97.3 | 83.6 |
| G04B | Urologicals | 72 | 10 | 3 | 15 | 7 | 2 | 109 | 98.2 | 91.7 |
| A06A | Drugs for constipation | 66 | 13 | 8 | 15 | 4 | 2 | 108 | 98.1 | 94.4 |
| C09A | ACE inhibitors, plain | 42 | 18 | 11 | 17 | 5 | 2 | 95 | 97.9 | 92.6 |
| B03A | Iron preparations | 65 | 13 | 5 | 3 | 5 | 2 | 93 | 97.8 | 92.5 |
| C03C | High-ceiling diuretics | 49 | 10 | 3 | 21 | 8 | 1 | 92 | 98.9 | 90.2 |
| A12A | Calcium | 46 | 26 | 3 | 6 | 4 | 1 | 86 | 98.8 | 94.2 |
| N05C | Hypnotics and sedatives | 19 | 3 | 1 | 34 | 17 | 10 | 84 | 88.1 | 67.9 |
| R06A | Antihistamines for systemic use | 50 | 15 | 1 | 11 | 2 | 3 | 82 | 96.3 | 93.9 |
| R03B | Other drugs for obstructive airway diseases, inhalants | 54 | 9 | - | 3 | 5 | 3 | 75 | 96.0 | 89.3 |
| V07A | All other non-therapeutic products | 55 | 6 | 1 | - | - | 1 | 63 | 98.4 | 98.4 |
| C03A | Low-ceiling diuretics, thiazides | 51 | 2 | - | 1 | 2 | 3 | 59 | 94.9 | 91.5 |
| G04C | Drugs used in benign prostatic hypertrophy | 36 | 15 | - | - | 5 | 1 | 57 | 98.2 | 89.5 |
| M05B | Drugs affecting bone structure and mineralisation | 24 | 23 | 1 | - | 4 | 3 | 55 | 94.5 | 87.3 |
| N05B | Anxiolytics | 20 | 3 | - | 17 | 9 | 6 | 55 | 89.1 | 72.7 |
| C03B | Low-ceiling diuretics, excluding thiazides | 35 | 10 | 1 | 1 | 1 | - | 48 | 100.0 | 97.9 |
| C01D | Vasodilators used in cardiac diseases | 19 | 9 | 1 | 10 | 7 | - | 46 | 100.0 | 84.8 |
| N05A | Antipsychotics | 27 | 2 | 2 | 11 | 3 | - | 45 | 100.0 | 93.3 |
| M04A | Antigout preparations | 24 | 10 | - | 5 | 1 | 2 | 42 | 95.2 | 92.9 |
| A03F | Propulsives | 34 | 1 | - | 4 | - | 1 | 40 | 97.5 | 97.5 |
| C09C | Angiotensin II receptor blockers (ARBs), plain | 12 | 10 | 2 | 6 | 6 | 3 | 39 | 92.3 | 76.9 |
| H02A | Corticosteroids for systemic use, plain | 14 | 1 | 3 | 11 | 4 | - | 33 | 100.0 | 87.9 |
| D07A | Corticosteroids, plain | 19 | 4 | - | 6 | 2 | - | 31 | 100.0 | 93.5 |
| C02C | Antiadrenergic agents, peripherally acting | 14 | 2 | 1 | 9 | 4 | - | 30 | 100.0 | 86.7 |
| V06D | Other nutrients | 24 | 3 | - | - | 1 | 2 | 30 | 93.3 | 90.0 |
| C01E | Other cardiac preparations | 15 | - | 2 | 6 | 3 | 1 | 27 | 96.3 | 85.2 |
| P01B | Antimalarials | 20 | - | 1 | 2 | 1 | 2 | 26 | 92.3 | 88.5 |
| N07C | Antivertigo preparations | 16 | - | - | 5 | 2 | - | 23 | 100.0 | 91.3 |
| N06D | Anti-dementia drugs | 9 | 1 | 1 | 3 | 5 | 2 | 21 | 90.5 | 66.7 |
| N04B | Dopaminergic agents | 6 | 2 | 4 | 4 | 5 | - | 21 | 100.0 | 76.2 |
| C03D | Aldosterone antagonists and other potassium-sparing agents | 14 | 2 | 1 | - | - | 2 | 19 | 89.5 | 89.5 |
| J01E | Sulfonamides and trimethoprim | 17 | - | - | - | 2 | - | 19 | 100.0 | 89.5 |
| H03A | Thyroid preparations | 1 | 4 | 9 | 3 | - | 1 | 18 | 94.4 | 94.4 |
| S01X | Other ophthalmologicals | 16 | - | - | - | - | - | 16 | 100.0 | 100.0 |
| C01B | Antiarrhythmics, class I and III | 8 | 1 | - | 4 | 1 | 1 | 15 | 93.3 | 86.7 |
| C09D | Angiotensin II receptor blockers (ARBs), combinations | 7 | 1 | 2 | - | 1 | 4 | 15 | 73.3 | 66.7 |
| A10A | Insulins and analogues | 4 | 2 | 4 | 3 | 2 | - | 15 | 100.0 | 86.7 |
| D02A | Emollients and protectives | 10 | 4 | - | - | - | - | 14 | 100.0 | 100.0 |
| R03D | Other systemic drugs for obstructive airway diseases | 11 | - | 1 | 1 | 1 | - | 14 | 100.0 | 92.9 |
| C04A | Peripheral vasodilators | 5 | 3 | 3 | 1 | 1 | 1 | 14 | 92.9 | 85.7 |
| N07B | Drugs used in addictive disorders | 12 | - | - | 1 | - | - | 13 | 100.0 | 100.0 |
| M02A | Topical products for joint and muscular pain | 9 | 3 | - | - | - | - | 12 | 100.0 | 100.0 |
| R01A | Decongestants and other nasal preparations for topical use | 9 | 3 | - | - | - | - | 12 | 100.0 | 100.0 |
| A11D | Vitamin B1, plain and in combination with vitamin B6 and B12 | 10 | 1 | - | - | - | 1 | 12 | 91.7 | 91.7 |
| D01A | Antifungals for topical use | 10 | 1 | - | - | 1 | - | 12 | 100.0 | 91.7 |
| C01C | Cardiac stimulants excluding cardiac glycosides | 5 | 1 | 2 | 3 | 1 | - | 12 | 100.0 | 91.7 |
| J01D | Other beta-lactam antibacterials | 7 | 2 | - | - | 1 | 2 | 12 | 83.3 | 75.0 |
| J01X | Other antibacterials | 11 | - | - | - | 1 | - | 12 | 100.0 | 91.7 |
| C09B | ACE inhibitors, combinations | 4 | 4 | - | - | 1 | 2 | 11 | 81.8 | 72.7 |
| N03A | Antiepileptics | 2 | - | 2 | 3 | 3 | - | 10 | 100.0 | 70.0 |
| A11A | Multivitamins, combinations | 7 | - | - | - | 2 | 1 | 10 | 90.0 | 70.0 |
| A03A | Drugs for functional gastrointestinal disorders | 8 | 1 | - | - | 1 | - | 10 | 100.0 | 90.0 |
| C01A | Cardiac glycosides | 4 | - | 1 | 4 | - | - | 9 | 100.0 | 100.0 |
| C08D | Selective calcium channel blockers with direct cardiac effects | 5 | - | - | 1 | 3 | - | 9 | 100.0 | 66.7 |
| A07D | Antipropulsives | 7 | - | 1 | 1 | - | - | 9 | 100.0 | 100.0 |
| D07C | Corticosteroids, combinations with antibiotics | 8 | - | - | - | 1 | - | 9 | 100.0 | 88.9 |
| A01A | Stomatological preparations | 6 | 2 | - | - | - | - | 8 | 100.0 | 100.0 |
| C03E | Diuretics and potassium-sparing agents in combination | 5 | - | - | 1 | 2 | - | 8 | 100.0 | 75.0 |
| M03B | Muscle relaxants, centrally acting agents | 6 | - | 1 | - | - | - | 7 | 100.0 | 100.0 |
| D06A | Antibiotics for topical use | 7 | - | - | - | - | - | 7 | 100.0 | 100.0 |
| S01K | Surgical aids | 4 | - | - | 1 | - | 1 | 6 | 83.3 | 83.3 |
| J01F | Macrolides, lincosamides and streptogramins | 3 | 1 | - | 1 | 1 | - | 6 | 100.0 | 83.3 |
| R05C | Expectorants, excluding combinations with cough suppressants | 3 | 2 | - | 1 | - | - | 6 | 100.0 | 100.0 |
| A07E | Intestinal anti-inflammatory agents | 4 | - | - | 3 | - | - | 6 | 100.0 | 100.0 |
| L01B | Antimetabolites | 4 | - | - | 1 | - | - | 5 | 100.0 | 100.0 |
| G03C | Oestrogens | 5 | - | - | - | - | - | 5 | 100.0 | 100.0 |
| L02B | Hormone antagonists and related agents | 4 | - | - | - | 1 | - | 5 | 100.0 | 80.0 |
| S01E | Antiglaucoma preparations and miotics | 3 | - | 1 | - | - | - | 4 | 100.0 | 100.0 |
| A03B | Belladonna and derivatives, plain | 4 | - | - | - | - | - | 4 | 100.0 | 100.0 |
| L04A | Immunosuppressants | 4 | - | - | - | - | - | 4 | 100.0 | 100.0 |
| A04A | Antiemetics and antinauseants | 2 | 1 | - | - | 1 | - | 4 | 100.0 | 75.0 |
| A12C | Other mineral supplements | 2 | 1 | - | - | - | - | 3 | 100.0 | 100.0 |
| J01A | Tetracyclines | 2 | - | 1 | - | - | - | 3 | 100.0 | 100.0 |
| A02A | Antacids | 2 | - | - | - | - | 1 | 3 | 66.7 | 66.7 |
| C02A | Antiadrenergic agents, centrally acting | 2 | - | - | - | 1 | - | 3 | 100.0 | 66.7 |
| G03X | Other sex hormones and modulators of the genital system | 2 | - | - | - | 1 | - | 3 | 100.0 | 66.7 |
| V03A | All other therapeutic products | - | 1 | - | - | 1 | - | 2 | 100.0 | 50.0 |
| D05A | Antipsoriatics for topical use | 1 | - | - | - | 1 | - | 2 | 100.0 | 50.0 |
| N04A | Anticholinergic agents | 1 | - | - | - | 1 | - | 2 | 100.0 | 50.0 |
| A07A | Intestinal anti-infectives | 1 | 1 | - | - | - | - | 2 | 100.0 | 100.0 |
| A11G | Ascorbic acid (vitamin C), including combinations | 2 | - | - | - | - | - | 2 | 100.0 | 100.0 |
| C05A | Agents for treatment of haemorrhoids and anal fissures for topical use | 2 | - | - | - | - | - | 2 | 100.0 | 100.0 |
| D10A | Anti-acne preparations for topical use | - | 2 | - | - | - | - | 2 | 100.0 | 100.0 |
| H03B | Antithyroid preparations | - | 1 | - | - | - | 1 | 2 | 50.0 | 50.0 |
| L01X | Other antineoplastic agents | 1 | 1 | - | - | - | - | 2 | 100.0 | 100.0 |
| N02C | Antimigraine preparations | 2 | - | - | - | - | - | 2 | 100.0 | 100.0 |
| G03B | Androgens | 1 | - | - | - | - | - | 1 | 100.0 | 100.0 |
| A11H | Other plain vitamin preparations | 1 | - | - | - | - | - | 1 | 100.0 | 100.0 |
| D08A | Antiseptics and disinfectants | 1 | - | - | - | - | - | 1 | 100.0 | 100.0 |
| D11A | Other dermatological preparations | 1 | - | - | - | - | - | 1 | 100.0 | 100.0 |
| G03F | Progestogens and oestrogens in combination | 1 | - | - | - | - | - | 1 | 100.0 | 100.0 |
| A05A | Bile therapy |  | - | 1 | - | - | - | 1 | 100.0 | 100.0 |
| A08A | Anti-obesity preparations, excluding diet products | 1 | - | - | - | - | - | 1 | 100.0 | 100.0 |
| A12B | Potassium | 1 | - | - | - | - | - | 1 | 100.0 | 100.0 |
| B03X | Other anti-anaemic preparations |  | - | 1 | - | - | - | 1 | 100.0 | 100.0 |
| C02D | Agents acting on arteriolar smooth muscle |  | - | - | 1 | - | - | 1 | 100.0 | 100.0 |
| D04A | Antipruritics, incl. Antihistamines, anaesthetics, etc. | 1 | - | - | - | - | - | 1 | 100.0 | 100.0 |
| D06B | Chemotherapeutics for topical use | 1 | - | - | - | - | - | 1 | 100.0 | 100.0 |
| G03D | Progestogens | 1 | - | - | - | - | - | 1 | 100.0 | 100.0 |
| L02A | Hormones and related agents | 1 | - | - | - | - | - | 1 | 100.0 | 100.0 |
| S01A | Anti-infectives | 1 | - | - | - | - | - | 1 | 100.0 | 100.0 |
| S01B | Anti-inflammatory agents | 1 | - | - | - | - | - | 1 | 100.0 | 100.0 |
| S01C | Anti-inflammatory agents and anti-infectives in combination | 1 | - | - | - | - | - | 1 | 100.0 | 100.0 |
|  | **Total** | **2522** | **847** | **240** | **746** | **359** | **152** | **4866** | **96.9** | **89.5** |

# **Appendix 3: Drug classes with within-class switch**

| **ATC 3^rd^ level code** | **ATC 3^rd^ level category** | **Number** |
| --- | --- | --- |
| A02B | Drugs for peptic ulcer and gastro-oesophageal reflux disease | 70 |
| A10B | Blood glucose lowering drugs, excluding insulins | 48 |
| C10A | Lipid modifying agents, plain | 43 |
| B01A | Antithrombotic agents | 40 |
| R03A | Adrenergics, inhalants | 20 |
| N06A | Antidepressants | 17 |
| A11C | Vitamin A and D, including combinations of the two | 15 |
| R06A | Antihistamines for systemic use | 10 |
| G04C | Drugs used in benign prostatic hypertrophy | 9 |
| N02A | Opioids | 8 |
| A12A | Calcium | 8 |
| M05B | Drugs affecting bone structure and mineralisation | 7 |
| M04A | Antigout preparations | 6 |
| A06A | Drugs for constipation | 5 |
| C09A | ACE inhibitors, plain | 5 |
| G04B | Urologicals | 5 |
| M01A | Anti-inflammatory and antirheumatic products, non-steroids | 5 |
| C03C | High-ceiling diuretics | 3 |
| C07A | Beta blocking agents | 3 |
| N02B | Other analgesics and antipyretics | 3 |
| N05C | Hypnotics and sedatives | 3 |
| C08C | Selective calcium channel blockers with mainly vascular effects | 2 |
| N05A | Antipsychotics | 2 |
| R03B | Other drugs for obstructive airways diseases, inhalants | 2 |
| A03F | Propulsives | 1 |
| A10A | Insulin and analogues | 1 |
| B03B | Vitamin B12 and folic acid | 1 |
| C03D | Aldosterone antagonists and other potassium-sparing agents | 1 |
| C04A | Peripheral vasodilators | 1 |
| C09B | ACE inhibitors, combinations | 1 |
| D01A | Antifungals for topical use | 1 |
| D02A | Emollients and protectives | 1 |
| D07A | Corticosteroids, plain | 1 |
| N04B | Dopaminergic agents | 1 |
| N05B | Anxiolytics | 1 |
| **Total** |  | **350** |

# **Appendix 4: Drug classes for ‘very significant’ (Eadon grade 5) interventions**

| **ATC 3rd level Classification** | | **Prescription altered stop**  **(*n*=439)** | **Prescription altered start**  **(*n*=54)** | **Prescription altered increase**  **(*n*=5)** | **Prescription altered decrease**  **(*n*=52)** | **Prescription endorsed**  **(*n*=2)** | **Patient/ carer education**  **(*n*=3)** | **Information given – patient**  **(*n*=1)** | **Information given – healthcare professional**  **(*n*=7)** | **Referral made**  **(*n*=9)** | **Requested/reviewed test, investigation,**  **or measurement**  **(*n*=4)** | **Other**  **(*n*=5)** | **Total**  **(*n*=581)** |
| --- | --- | --- | --- | --- | --- | --- | --- | --- | --- | --- | --- | --- | --- |
| B01A | Antithrombotic agents | 113 | 17 | 3 | 8 | - | - | - | - | 1 | - | - | 142 |
| M01A | Anti-inflammatory and antirheumatic products, non-steroids | 116 | - | - | 1 | - | - | - | - | - | - | - | 117 |
| - | Not specified | 6 | - | - | - | 1 | 3 | - | 7 | 8 | 4 | 5 | 34 |
| A10B | Blood glucose lowering drugs, excluding insulins | 21 | 2 | 1 | 3 | - | - | - | - | - | - | - | 27 |
| C07A | Beta blocking agents | 15 | - | - | 10 | - | - | - | - | - | - | - | 25 |
| N06A | Antidepressants | 16 | - | - | 4 | - | - | - | - | - | - | - | 20 |
| A02B | Drugs for peptic ulcer and gastro-oesophageal reflux disease | 2 | 16 | - | 1 | - | - | - | - | - | - | - | 19 |
| C09A | Ace inhibitors, plain | 11 | - | - | 3 | - | - | - | - | - | - | - | 14 |
| N02B | Other analgesics and antipyretics | 10 | - | - | 3 | - | - | 1 | - | - | - | - | 14 |
| A03F | Propulsives | 11 | - | - | - | - | - | - | - | - | - | - | 11 |
| G04B | Urologicals | 11 | - | - | - | - | - | - | - | - | - | - | 11 |
| N02A | Opioids | 9 | - | - | 2 | - | - | - | - | - | - | - | 11 |
| N05A | Antipsychotics | 9 | - | - | 1 | - | - | - | - | - | - | - | 10 |
| C03A | Low-ceiling diuretics, thiazides | 9 | - | - | - | - | - | - | - | - | - | - | 9 |
| C03B | Low-ceiling diuretics, excluding thiazides | 9 | - | - | - | - | - | - | - | - | - | - | 9 |
| H02A | Corticosteroids for systemic use, plain | 7 | - | - | 2 | - | - | - | - | - | - | - | 9 |
| N05B | Anxiolytics | 6 | - | - | 3 | - | - | - | - | - | - | - | 9 |
| C10A | Lipid modifying agents, plain | 1 | 5 | 1 | 1 | - | - | - | - | - | - | - | 8 |
| C09C | Angiotensin II receptor blockers (ARBs), plain | 6 | - | - | 1 | - | - | - | - | - | - | - | 7 |
| C03D | Aldosterone antagonists and other potassium-sparing agents | 6 | - | - | - | - | - | - | - | - | - | - | 6 |
| C08D | Selective calcium channel blockers with direct cardiac effects | 4 | - | - | 1 | - | - | - | - | - | - | - | 5 |
| R03D | Other systemic drugs for obstructive airway diseases | 4 | - | - | 1 | - | - | - | - | - | - | - | 5 |
| A12A | Calcium | 1 | 3 | - | - | - | - | - | - | - | - | - | 4 |
| R06A | Antihistamines for systemic use | 4 | - | - |  | - | - | - | - | - | - | - | 4 |
| C01A | Cardiac glycosides | 1 | - | - | 2 | - | - | - | - | - | - | - | 3 |
| C01D | Vasodilators used in cardiac diseases | 1 | 2 | - | - | - | - | - | - | - | - | - | 3 |
| C02C | Antiadrenergic agents, peripherally acting | 1 | 2 | - | - | - | - | - | - | - | - | - | 3 |
| G04C | Drugs used in benign prostatic hypertrophy | 3 | - | - | - | - | - | - | - | - | - | - | 3 |
| J01E | Sulfonamides and trimethoprim | 3 | - | - | - | - | - | - | - | - | - | - | 3 |
| C01B | Antiarrhythmics, class I and III | 1 | - | - | 1 | - | - | - | - | - | - | - | 2 |
| C01E | Other cardiac preparations | 2 | - | - | - | - | - | - | - | - | - | - | 2 |
| C03C | High-ceiling diuretics | 2 | - | - | - | - | - | - | - | - | - | - | 2 |
| C08C | Selective calcium channel blockers with mainly vascular effects | 2 | - | - | - | - | - | - | - | - | - | - | 2 |
| C09B | ACE inhibitors, combinations | 2 | - | - | - | - | - | - | - | - | - | - | 2 |
| M03B | Muscle relaxants, centrally acting agents | 2 | - | - | - | - | - | - | - | - | - | - | 2 |
| M04A | Antigout preparations | 1 | - | - | 1 | - | - | - | - | - | - | - | 2 |
| M05B | Drugs affecting bone structure and mineralisation | - | 2 | - | - | - | - | - | - | - | - | - | 2 |
| N03A | Antiepileptics | 1 | - | - | 1 | - | - | - | - | - | - | - | 2 |
| N04B | Dopaminergic agents | 2 | - | - | - | - | - | - | - | - | - | - | 2 |
| N06D | Anti-dementia drugs | 1 | - | - | - | 1 | - | - | - | - | - | - | 2 |
| A01A | Stomatological preparations | 1 | - | - | - | - | - | - | - | - | - | - | 1 |
| A07E | Intestinal anti-inflammatory agents | - | - | - | 1 | - | - | - | - | - | - | - | 1 |
| A10A | Insulins and analogues | - | 1 | - | - | - | - | - | - | - | - | - | 1 |
| A11C | Vitamin A and D, including combinations of the two | 1 | - | - | - | - | - | - | - | - | - | - | 1 |
| C01C | Cardiac stimulants excluding cardiac glycosides | - | 1 | - | - | - | - | - | - | - | - | - | 1 |
| C09D | Angiotensin II receptor blockers (ARBs), combinations | 1 | - | - | - | - | - | - | - | - | - | - | 1 |
| G03B | Androgens | 1 | - | - | - | - | - | - | - | - | - | - | 1 |
| H03A | Thyroid preparations | - | 1 | - | - | - | - | - | - | - | - | - | 1 |
| L01B | Antimetabolites | - | - | - | 1 | - | - | - | - | - | - | - | 1 |
| L02A | Hormones and related agents | 1 | - | - | - | - | - | - | - | - | - | - | 1 |
| N04A | Anticholinergic agents | 1 | - | - | - | - | - | - | - | - | - | - | 1 |
| N05C | Hypnotics and sedatives | 1 | - | - | - | - | - | - | - | - | - | - | 1 |
| R03A | Adrenergics, inhalants | - | 1 | - | - | - | - | - | - | - | - | - | 1 |
| V07A | All other non-therapeutic products | - | 1 | - | - | - | - | - | - | - | - | - | 1 |
